# Bioprospective Role of *Ocimum sanctum* and *Solanum xanthocarpum* against Emerging Pathogen: *Mycobacterium avium* Subspecies *paratuberculosis*: A Review

**DOI:** 10.3390/molecules28083490

**Published:** 2023-04-15

**Authors:** Manthena Nava Bharath, Saurabh Gupta, Garima Vashistha, Sayeed Ahmad, Shoor Vir Singh

**Affiliations:** 1 Department of Biotechnology, Institute of Applied Science & Humanities, GLA University, Mathura 281406, India; saurabh.gupta@gla.ac.in (S.G.); garimavashistha2001@gmail.com (G.V.); 2 Bioactive Natural Product Laboratory, Centre of Excellence in Unani Medicine (Pharmacognosy and Pharma Cology), School of Pharmaceutical Education and Research, Jamia Hamdard, New Delhi 110062, India; sahmad_jh@yahoo.co.in

**Keywords:** bioactive compounds, *Mycobacterium avium* subspecies *paratuberculosis* (MAP), *Solanum xanthocarpum*, *Ocimum sanctum*, diagnosis, PCR, ELISA

## Abstract

*Mycobacterium avium* subspecies *paratuberculosis* (MAP) is a chronic, contagious, and typically life-threatening enteric disease of ruminants caused by a bacterium of the genus Mycobacterium, but it can also affect non-ruminant animals. MAP transmission occurs through the fecal–oral pathway in neonates and young animals. After infection, animals generate IL-4, IL-5, and IL-10, resulting in a Th2 response. Early detection of the disease is necessary to avoid its spread. Many detection methods, viz., staining, culture, and molecular methods, are available, and numerous vaccines and anti-tuberculosis drugs are used to control the disease. However, the prolonged use of anti-tuberculosis drugs leads to the development of resistance. Whereas vaccines hamper the differentiation between infected and vaccinated animals in an endemic herd. This leads to the identification of plant-based bioactive compounds to treat the disease. Bioactive compounds of *Ocimum sanctum* and *Solanum xanthocarpum* have been evaluated for their anti-MAP activity. Based on the MIC50 values, Ursolic acid (12 µg/mL) and Solasodine (60 µg/mL) were found to be suitable for anti-MAP activity.

## 1. Introduction

**Figure 1:**
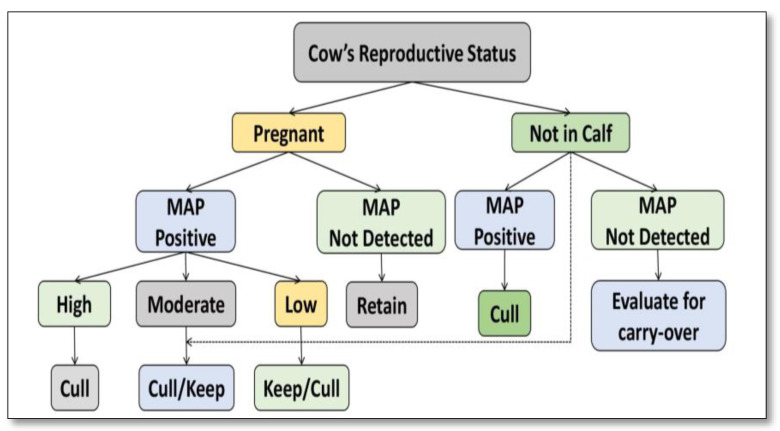
Control of *Mycobacterium avium* subspecies *paratuberculosis* (MAP) infection in domestic livestock.

Paratuberculosis (Johne’s disease) is caused by *Mycobacterium avium* subspecies *paratuberculosis* (MAP), a highly resistant, extremely fastidious, Gram-positive acid-fast bacilli. The disease is characterized by reduced productivity, loss in body weight, and can be experienced with (in cattle and buffaloes) or without (in goats and sheep) diarrhea. Animals acquire infection in the early stages (prenatal) through semen or in utero, with colostrum and milk, or from the environment after birth via the fecal–oral route. The high endemicity of MAP in domestic livestock leads to high propagation in herds and flocks. As it is highly resistant in a few bacilli, MAP requires multidrug therapy for a long time (7–8 months), which is not cost-effective even when the cost of animals is not considered. Johne’s disease in ruminants is caused by *Mycobacterium avium* subsp. *paratuberculosis* (MAP), a persistent bacterium with significant economic effects and global dissemination [1]. The apparent correlation between *Mycobacterium avium* subspecies *paratuberculosis* and Crohn’s disease in individuals is still being studied extensively, with conflicting results [2,3,4]. In 1895, German researchers Johne and Frothingham acknowledged MAP for the first time [5]. As shown in Figure 1, it commonly infects ruminants (cattle, sheep, goats, deer, etc.), but it has also been reported in non-ruminants, notably wildlife [6]. Annual cattle sector losses in the United States are estimated to be between $250 million [7] and $1.5 billion [8]. According to a new assessment of available data, which employed a Bayesian technique [9] calibrated for susceptivity and explicitness, the underlying frequency of MAP in dairy cattle in the United States was 91.1%, not the 70.4% claimed in 2007 [10]. The incidence of MAP in beef cattle herds is 7.9% [11]. Even though JD was initially discovered in the United States during the early 1900s, the emphasis on investigation and disease prevention alone has expanded in the last 20 years. To combat Johne’s disease on a farm and recognize herds with minimal infection susceptibility, a discretionary Bovine JD Management Program is in operation. The examination of ambient stool specimens via culturing through elevated sites is among the most cost-effective and highly reliable diagnostic techniques for JD [9]. Ironically, wildlife repositories may disrupt initiatives to reduce Johne’s disease in livestock unless their significance in wildlife is completely defined [12]. JD transmission is reduced when improved diagnoses are combined with good management strategies [13].

The present pilot study aims to evaluate the in vitro anti-mycobacterial activity of bioactive constituents from *Ocimum sanctum* and *Solanum xanthocarpum* plants. The Resazurin microtiter plate assay (REMA)was performed to investigate the activity of two bioactive compounds against MAP. Experimental studies confirmed the anti-MAP potential of Ursolic acid and solasodine, which were found to be the best bioactive compounds (BAC) inhibiting the bacilli. The activity was expressed as MIC 50 values of Ursolic acid (12 µg/mL) and Solasodine (60 µg/mL). This is the first attempt to suggest a potential therapeutic value against the highly pathogenic native ‘S 5′ strain of MAP, giving a ray of hope for the development of a herbal anti-MAP medication for domestic livestock, which could pave the way for the development of therapy in human beings since it is a major infection associated with autoimmune disorders.

## 2. Taxonomy and Properties

The *Mycobacterium avium* complex, which belongs to the genus *Mycobacterium* and the family Mycobacteriaceae, contains MAP. *Mycobacterium avium* and *Mycobacterium intracellulare* are two distinct species in the *Mycobacterium avium* complex. *Mycobacterium avium* subsp. *avium*, *Mycobacterium avium* subsp. *hominissuis* (MAH), MAP, and *Mycobacterium avium* subsp. *silvaticum* are the four subspecies of *M. avium*, according to a thorough sequence-based evaluation of the internal transcribed spacer of 16S-23S ribosomal RNA [14,15]. MAP is a Gram-positive, acid-fast, rod-shaped intracellular bacteria with a diameter of 0.5 to 1.5 m. The bacterial cell wall is dense and waxy arabinogalactan holds the mycolate and peptidoglycan layers intact. Bacteria are slow-growing and take over 20 h to multiply [16]. Efforts to build up MAP in the research lab medium were initially unsuccessful [17], and it was hypothesized that MAP’s failure to cultivate in vitro was due to the lack of a crucial development factor.

Further analysis revealed that MAP could flourish on a medium enriched with extracts from many other mycobacteria [18,19], leading scientists to assume that MAP cannot generate the vital growth factor that some other species can synthesize. Mycobactin is a siderophore that binds iron and is produced from Mycobacterium phlei, which has been identified as the growth factor required for MAP cultivation in vitro [20,21]. Mycobactin dependence has been regarded as taxonomic for MAP since that period. A mutation in the mbtA gene in the mycobactin-production operon has recently rein forced the molecular knowledge of mycobactin reliance, as explained in the genome sequence below [22,23].

## 3. Pathogenesis

**Figure 2:**
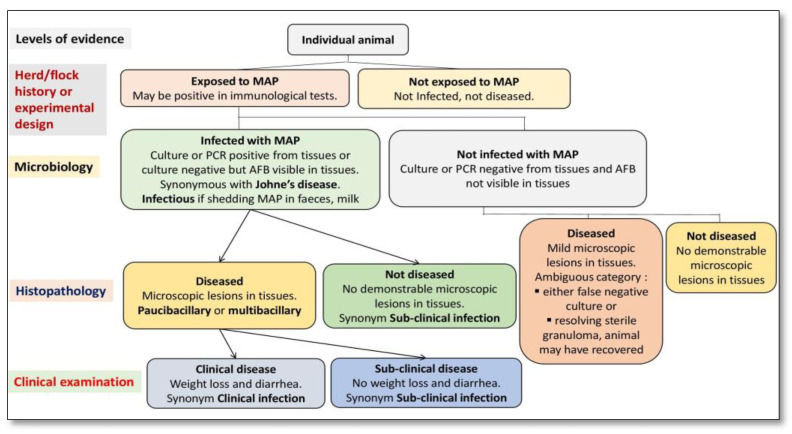
Primary classification of animals exposed to *Mycobacterium avium* subspecies *paratuberculosis* (MAP) using a systematic diagnostic approach.

Johne’s disease (JD) is characterized by persistent diarrhea and a mal absorption condition, which results in malnutrition and muscle atrophy (Figure 2). The fecal–oral pathway is the most common way for neonates and young animals to become infected. Milk feeding from an infected dam is another source of infection to neonates [24]. Calves up to the age of six months have a greater incidence of infection, but afterwards, the risk reduces [25]. According to animal research, M-cells and enterocytes both promote MAP adjunct to and transit through the gut mucosa upon consumption [26]. Tissue culture observations demonstrate that MAP influences the establishment of tight junctions in the intestinal mucosa, offering a mechanism for enhanced permeability [27]. Antigens 85 [28], 35 kDa [29], MAP oxidoreductase [30], MAP fibronectin-binding protein [31,32], and histone HupB [33] are all crucial in MAP epithelial cell adhesion and/or penetration, and host–pathogen interaction occurs consistently.

**Figure 3:**
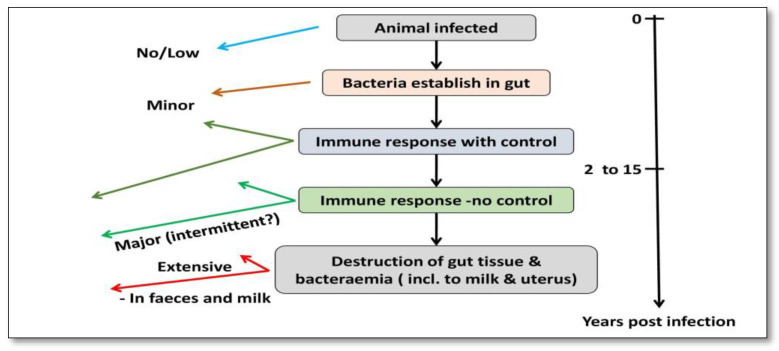
Pathogenesis of MAP infection in cattle.

The prior literature has shown that phagosome acidification stimulates interleukin (IL)-1 production, macrophage recruitment, and trans-epithelial migration in MAP-infected epithelial cells, utilizing the cow mammary epithelial cell line MAC-T [34] and bovine blood-monocyte-derived macrophages (BMDM) [35]. Bacilli (genus *Bacillus*) are subsequently phagocytosed in the sub- and intraepithelial spaces by these macrophages [36,37,38]. For pathogenesis, MAP’s capacity to persist and proliferate once inside phagocytic cells is fundamental [39,40]. Furthermore, researchers observed that the lipid content of MAP changes in macrophages that acquire a pro-inflammatory phenotype utilizing a culture passage model [41] (Figure 3).

The pathognomonic granulomatous enteritis of Johne’s illness [38], which is characterized by a wide and ridged intestinal wall as well as inflammatory lymph nodes, is the result of the ensuing host cellular immunological response. Toll-like receptors help tissue macrophages and dendritic cells recognize molecular patterns linked with pathogens in the innate phase, as well as the abstraction of cytokine-mediated cellular connections and antigen processing [42,43]. In the acquired immunity phase, Th1 T-helper cell responses and the concurrent stimulation of macrophages by interferon-gamma (INF) produced by Th1 T cells are used to reduce MAP infections. [44,45]. The inferential function of nitric oxide synthase has already been shown in cattle and is implicated in the killing process of these activated phagocytic cells [46].

**Figure 4:**
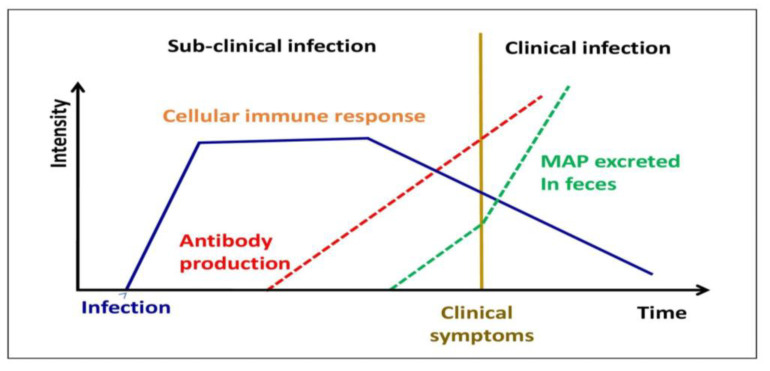
Different stages of MAP infection in domestic livestock.

In this condition, BMDM recovered from sub-clinically contaminated animals exhibits exceptionally high levels of nitric oxide generation [47] (Figure 4). MAP, on the other hand, affects the activity of bovine macrophages, as demonstrated by distinct profiles of mRNA expression [48], apoptosis suppression and antigen distribution [49], and diagnostic cytokine expression patterns [50]. In infected bovine T helper cells, MAP mostly generates a Th2 response, with an increase in production of IL-4, IL-5, IL-10, and tissues remodeling inhibitors [51,52]. This humoral response was confirmed in a newborn calf model [53]. In addition, in both ruminants and animals, regulatory T and Th17 cells have been involved in the immune pathogenesis of JD [49,54].

**Figure 5:**
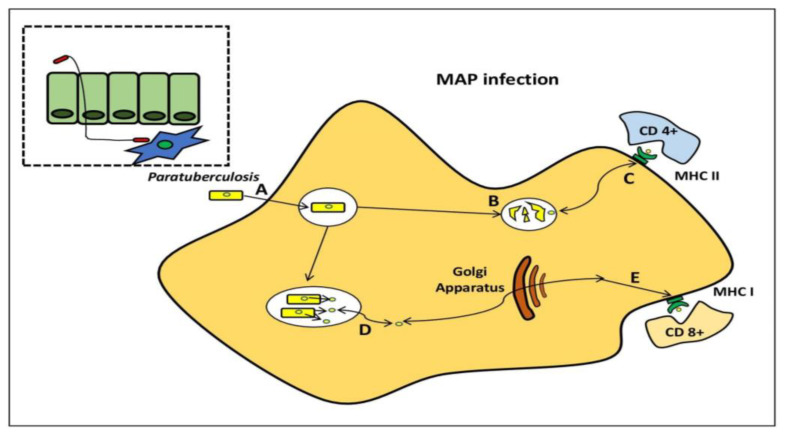
*Mycobacterium avium* subspecies *paratuberculosis* within the macrophage. MAP bacilli pass by the mucosal barrier, preferentially via M cells, and after which it is engulfed by sub-epithelial macrophage (A). MAP bacilli degrade within the phagosome (B) and also stimulateCD4+ immune responses via antigen presentation through the MHC class II pathway. (C) MAP evades destruction within phagosome through the inhibition of general killing mechanisms and proliferates. (D) MAP proteins are secreted from the phagosome to the cytosol and are subsequently available for presentation to (E) CD8+ immune responses via the MHC class I pathway.

MAP pathogenesis has been studied using a variety of models. MAP, on the other hand, produces immunological responses in ruminant hosts, which are not found in traditional in vitro models. MAP bacilli grow during 4–8 days in infected BMDM [44,55], although bacterial burdens are reduced over time after infection of the murine J774 macrophage cell line [44,55,56,57]. When researching, the interactions between MAP and phagocytic cells, it is preferable to use primary phagocytic cells. To follow the progression of MAP infection from the initial to final stages, Ileal loops have been employed to establish a prospective systems biology approach [58]. The host transcriptome profile following infection with *M. avium* subsp. *avium* and MAP were recently compared using this paradigm. Intestinal mucosal weakening, activation of a Th2 reaction, and phagocytosis suppression were all related to MAP transmission, which was not found with *M. avium* subsp. *avium* infection [59] (Figure 5).

## 4. Diagnosis and Control

Before any clinical indications, infected animals shed MAP in their feces, making them a prominent cause of infection for the herd’s other animals. To avoid the spread of JD, it is critical to diagnose the infection as soon as possible. Based on the detection of MAP both directly and indirectly, many diagnostic tests have been created [60]. The direct identification of MAP in clinical specimens can be achieved using (i) microscopy, (ii) culture-based MAP isolation, and (iii) PCR-based MAP DNA identification. Clinical samples have been analyzed using acid-fast or Ziehl–Neelsen staining. Acid-fast staining is the easiest, quickest, and most economical mode of diagnosis, but its accuracy and precision are inadequate since it is challenging to discern between MAP and some other acid-fast bacilli [61].

Although Ziehl–Neelsen staining can also be used to screen for MAP, it must be verified by additional procedures such as PCR and/or immunoassays. The “gold standard” for JD diagnosis is MAP isolation through culture. The fact that MAP requires mycobactin J to grow in a specific laboratory medium can be utilized to distinguish it from many other acid-fast bacteria. A novel growth media that increases MAP restoration and sensitivity by 1000-fold was recently divulged [62]. Due to the fact that MAP develops slowly (On solid medium, colony development takes 6–8 weeks.), culture-based diagnosis takes a long period. Consequently, a highly fast and precise PCR-based test was employed for MAP identification in environmental and clinical specimens [63,64,65]. *IS900* is a 1.4 kb multi-copy insertion element that is sequence-specific to MAP. The primers used in this PCR are for *IS900* [60,66]. Other *mycobacteria* with *IS900*-like insertion sequences, on the other hand, have been demonstrated to influence the specificity of this test, resulting in false-positive findings [64,67].

To prevent false-positive results, a multiplex PCR centered on the *IS900*, *IS901*, *IS1245*, and dnaJ genes was constructed, although the precision of this assay is restricted due to the reagent interference and primer-dimer generation [60,68]. Furthermore, PCR tests based on stool specimens hold only 70% sensitivity and 85% specificity [69]. There has been some advancement in identifying and utilizing more precise targets for PCR testing [70,71,72], and this comparative genomic technique has addressed an apprehension gap in MAP identification. Several of these objectives have made their way into commercial diagnostic tools. The immunological response of the host to infection is the basis for diagnostic MAP tests based on indirect detection. A Johnin pure protein derivative was used to produce the delayed-type hypersensitivity skin test [73].

However, because various environmental mycobacteria might sensitize the animal and provide false-positive findings, this test is not specific. As a result, delayed-type hypersensitivity skin tests cannot tell the difference between vaccinated animals and those that have been naturally affected. As previously established, MAP invasion triggers T helper cells, which secrete IFN-γ.The utilization of cultures with supernatants from day-old blood specimens which are treated with Johnin and co-stimulated with human IL-2and/or bovine IL-12 can also be used to diagnose JD using an enzyme-linked immune sorbent test (ELISA) [74]. Unfortunately, cross-reactivity issues arise because, in the INF-test, MAP pure proteins analogs are often used as antigens. A potential alternative MAP antigen for the research was L5P, a cell wall lipopeptide; however, the IFN-γ expression was reported to be weaker than that of Johnin [75].

Antibodies in milk and serum from diseased animals are detected using commercial ELISA kits such as (I)) ParaCheck (CSL/Biocor), (ii) HerdCheck *M. paratuberculosis* ELISA (IDEXX Laboratories, Inc., Westbrook, ME, USA), (iii) ID Screen^®^*Paratuberculosis* Indirect (ID Screen^®^*Paratuberculosis* Indirect (ID Screen (IDvet Genetics)and (iv) SERELISA *ParaTB* (Synbiotic Corp., Kansas City, MO, USA). In comparison to PCR testing, an ELISA seems to have a lower sensitivity of 50% but a far higher specificity of 99.8% [76,77]. To establish better sensitive immune-based tests for JD diagnosis, additional research is needed to uncover specialized MAP antigens. Vaccination (the most economical), screening, and improved herd control are all alternatives for avoiding JD, depending on a producer’s finances, infrastructure, and operations [78]. However, while JD vaccines can diminish systemic disease and discharge, their effectiveness is minimal, and none of them provide fairly long immunity.

In the United States, for instance, Mycopar^®^ (BoehringerIngelheimVetmedica, Inc., St Joseph, MO, USA) has been the exclusively licensed vaccination for JD in cattle. Unfortunately, since strain 18 of *M. avium* subsp. *avium* was used to make the vaccine [79], it lacks an ideal antigenic repertoire. In Australia, Silirum^®^ (Zoetis Animal Health, Parsippany-Troy Hills, NJ, USA), a different bacterin, is being investigated, and it has been licensed for restricted usage in cattle. The MAP 316F strain has been heat-killed in this vaccination. This formulation may contain a broader spectrum of antigenic; however, utilizing bacteria that have been destroyed by heat may lower efficacy while improving safety. Both Neoparasec^®^ (Rhone-Merieux, Athens, GA, USA) and Gudair^®^ (Zoetis Animal Health) contain the live-attenuated MAP strain 316F and are authorized for usage in goats and sheep. Vaccines that are currently available, on the other hand, are unable to discriminate between vaccinated and infected animals, impairing JD diagnostic testing [80], and strain 316F was created in the 1920s using random depreciation processes (e.g., passages in ox bile) that are currently being examined [81]. Eventually, to successfully manage JD, an elevated vaccination is necessary [82].

The latest batch of human anti-tuberculosis vaccines appear to provide better protection than subunit vaccines, according to testing results [83]. Because JD is induced by a bacteria called Mycobacterium, potential subunit or bacterin-based vaccines are likely to face a similar situation. The JD Integrative Protocol-Animal and Plant Health Inspection Service’s endeavors to establish a consistent vaccination testing program were spurred by this. In a three-phase investigation, investigators from New Zealand and the U.S provided 22 masked live-attenuated immunization candidates to be evaluated in mouse, BMDM, and goat models. Despite the substantial development of animal screening procedures [84], the bulk of the suppressed transposon variants investigated were from the first generation and had the Tn5367 transposase, causing destabilization. Furthermore, unknowns, including the ideal immunization path and dose plan, could not be determined before the commencement of the experiment. Despite this, crucial information and chemicals were created [80]. The design of a subunit vaccine that can manage infections by inducing the appropriate humoral immunity [85], specifically against antigens produced by the pro-inflammatory phenotype is not yet conceivable [86].

However, in the absence of a vaccine, the control of MAP infection in the human population can be accomplished either via the surgical removal of infected intestines or by medicines [87] using anti-tuberculosis drugs, which have had limited success [88,89]. The prolonged use of anti-tuberculosis drugs has resulted in drug resistance to all of the existing anti-mycobacterial molecules. Because of the increase in cases of animal and human infections, the demand for natural products as an alternative therapy for this chronic incurable disease has increased. This has encouraged researchers to find out bio-active (marker) compounds from plants with pharmacological properties against symptoms exhibited by MAP-infected domestic livestock populations, e.g., chronic progressive inflammation, etc. Prior studies have suggested that plant extracts can feasibly decrease the induction of TNF-α that modulates TNF-α mediated inflammatory pathways, and this may have potential against diseases arising due to chronic inflammation caused by MAP infection (paratuberculosis or Johne’s disease in animals and Crohn’s disease in humans). Plant extracts play major roles as immuno-modulators and immuno-stimulators and can increase or decrease the level of various pro-inflammatory and inflammatory cytokines during chronic inflammation.

The pre-2nd century ‘Charaka Samhita’ book reported that Ayurveda (Indian traditional medicine) herbal medicinal plants have been used to cure tuberculosis and other various ailments. Decoctions, Infusions, Tinctures, and macerations of herbal medicinal plant parts such as fruits and flowers, stem bark, roots, stems, and leaves have been used as part of traditional treatments for many centuries by native people worldwide. Even though ethnopharmacological and ethnobotanical studies have considered the wide use of herbal medicinal plants in the treatment of TB, most of them were established still to be therapeutic and safe doses. Most research studies have failed to give scientific proof to therapeutic practices and traditional beliefs. Consequently, this work aims to archive the traditional medicinal plants used to control TB and contrast the traditional therapeutic systems that have been used to cure TB, from the poorly documented oral Indian medicines to the well-documented Indian Ayurveda and so on.

## 5. Description of *Ocimum sanctum* Plant

### 5.1. Taxonomic Classification of Ocimum sanctum Plant

Scientific Name: *Ocimum sanctum*

Ayurvedic Name: Tulsi

Division: Magnoliophyta

Class: Magnoliopsida

Subclass: Asteridae

Order: Lamiales

Family: Lamiaceae

Genus: Ocimum

### 5.2. Morphology

Tulsi (*Ocimum sanctum*) is an upright, multi-branched sub-shrub, which is approximately 300–600 mm (30–60 cm) tall with hairy stems. The colors of its leaves are purple or green, and the petiole is up to 5 cm (2 inches) long; it has an ovate blade and also a slightly toothed margin. The plant fragrant is very strong and Phyllotaxy is decussate. The flowers are purplish and placed in close whorls on elongated racemes [90]. In India and Nepal, three main types of morphotypes are cultivated: Ram tulsi(which is a common type with broad bright slightly sweet green leaves), Krishna or Shyam tulsi (in which purplish green leaves are less common), and Vana tulsi (which is most commonly found in the wild) [91].

### 5.3. Soil and Climate

*Ocimum sanctum* (Holy basil) plant can be grown in moderately shaded conditions with low oil contents. Waterlogged conditions can cause root rot and stunt growth. *Ocimum sanctum* flourishes under high rainfall and humid conditions. The high temperatures and long days have been proven to be favorable for plant growth and oil production. Soil and manure—preferably porous, aerated, and well-drained—with added organic manure and soil is required for plant growth. Clay and sticky soil is not good for the plant’s roots.

### 5.4. Floral Characteristics

*Ocimum sanctum* plant is a short-lived perennial shrub or small annual, which can reach up to 3.3 feet (100 cm) in height. They have hairy simple-toothed stems. The scented leaves are purple or green, depending on the variety. The white tubular or small purple flowers have green or purple sepals and are supported by terminal spikes.

### 5.5. Propagation

*Ocimum sanctum* crops can be propagated through seeds beings own in nursery beds. In total, 300g of seeds are required in one hectare for the sowing. The nursery should be located in partial shade with sufficient irrigation facilities with a soil depth up to 30 cm. Organic manure is applied to the soil and prepared so that the size of the seed beds are 4.5 × 1.0 × 0.2 m. The quantity of seeds is mixed with the sand in a 1:4 ratio. For seeds sown a nursery bed, this is required 60 days in advance of the onset of a monsoon. The 8–12-day-old seeds can germinate and transplant seedlings for about 6 weeks during the 4–5 leaf stage.

### 5.6. Distribution

The Holy basil plant is widely distributed throughout India, and researchers from the Central University of Punjab and Bathinda have studied the large-scale phylogeny graphical of this species using chloroplast whole genomic sequencing, which revealed that this holy basil plant originated from North-Central India [92].

### 5.7. Medicinal Uses of Bioactive Compounds

*Ocimum sanctum* is a native herb in India. Also known as ‘Tulsi,’ it belongs to the Lamiaceae family. The Hindu religious tradition is sacred, and Tulsi is perhaps viewed as the most significant plant used in Ayurvedic medicine [93]. Tulsi plants grow in abundance around Hindu temples and can be found in multiple varieties, e.g., green, red, or with a strong but pleasant aroma. In the previous decade, several shreds of scientific evidence have reported [94,95,96] that holy basil has been utilized to treat a variety of many critical diseases [97], including asthma, arthritis, heart problems, eye disorders, blood glucose levels, hepato protective, anticancer, anti-fungal, antimicrobial, chronic fever, anti-fertility, and bronchitis [98,99] (Table 1). *Ocimum sanctum* has many chemical constituents, such as carvacrol, eugenol, limatrol, linalool, ursolic acid, caryophyllene, propionic acid, methyl carvicol, Rosmarinic acid, Apigenin, cirsimaritin, Orientin, isothymusin, and Vicenin. Supplementary materials Figures S1 and S2 show the biological mechanism between the a fore mentioned bioactive constituents and their MIC50 values are given in Table 1. Previous research has also showed that the leaf juice extracted from Tulsi demonstrates the inhibition of complete growth in anti-viral and anti-mycobacterial activities [100,101].

## 6. Description of *Solanum xanthocarpum* Plant

### 6.1. Taxonomic Classification of Solanum xanthocarpum Plant

Scientific Name: Solanum xanthocarpum

Ayurvedic Name: Kantakari

Division: Magnoliophyta

Class: Magnoliopsida

Subclass: Asteridae

Order: Solanales

Family: Solanaceae

Genus:Solanum

### 6.2. Morphology

*Solanum xanthocarpum* plant is a very thorny diffused bright green perennial herb and, at the base, it is woody. Its many branches spread on the ground, and the newest branches are covered with dense stellate tomentum, yellow, straight, glabrous, prickles compressed, shining often exceeding and 13 mm long. Its leaves are 50–100 × 25–57 mm, bearing stellate hairs on both sides or beneath (ovate or elliptic) the petioles, which are 13–25 mm long, sometimes becoming nearly glabrous with age.

### 6.3. Soil and Climate

*Solanum xanthocarpum* is a hardy plant mainly grown in tropical and sub-tropical regions. It does adequately over light humus-rich, silty sand to enrich loamy soils with a pH of 7.0–8.0. Kantakari is a warm-season crop grown over saline lands. The most favorable temperature range for its growth and reproduction is 21–27 °C. Generally, abundant sunshine and dry weather is required. In northern India, from December to January, this crop is adversely affected due to frost, as it causes injury to vegetative parts. In the spring, the plant recovers.

### 6.4. Floral Characteristics

Kantkari flowers have axillary bud/cymes and a bluish-violet color. The curved hairy stellate with short pedicels, linear-lanceolate, globose, and prickly outside lobes are 1.1 cm long. They have purple Cololla lobes that are 20 mm long, acute, and hairy on the outside. They have 1.5 mm of Filament, 8 mm of anthers, and glabrous, oblong-lanceolate tiny pores that open up. Style glabrous and ovary is ovoid. The berry-shaped fruits, approximately 13–20 mm in diameter, are white or yellow with green veins, and the calyx is enlarged. Seeds are typically 2.5 mm in diameter, in sub-reni form, glabrous, smooth, and yellowish-brown.

### 6.5. Distribution

Kantakari plant is widely distributed throughout India, particularly due to the dry climate in the Himalayas, where weeds can ascend up to heights of 1500 m. Kantakari plants grow in abundance by roadsides and wastelands, mainly in Uttar Pradesh, Rajasthan, Madhya Pradesh, Gujarat, and Haryana.

### 6.6. Propagation

The crop is elevated via sowing seeds with a Yellowish-brown color and small size, i.e., 0.25 cm in diameter and glabrous. There is no dormancy period for the seeds, and they can be sown after a few days of harvesting. The germination percentage is around 60–70%, and they take 10–15 days to germinate.

### 6.7. Medicinal Uses of Bioactive Compounds

*Solanum xanthocarpum* is a native herb of India. Also known as Kantakari, it belongs to the Solanaceae family. It is a thorny, bright green, perennial plant with woody roots that grow to a height of 2 to 3 m and is found all over India, primarily in arid regions such as highway shoulders and wastelands.They produce expanded calyx-shaped fruits which appear as yellow or white berries with green veins and have a diameter of 1.3 cm [112]. In the previous decade, many scientific studies have reported [113] that Kantakari has been utilized to treat a variety of many critical diseases, including cough, fever, heart diseases, antipyretic, hypotensive, antiasthmatic, antitumor, anti-anaphylactic, aphrodisiac activities, wound healing, anti-inflammatory, urinary bladder, laxative-use [114], blood glucose levels, hepatoprotective, anticancer, antifungal, antimicrobial, chronic fever, antifertility, and bronchitis [115].

*Solanum xanthocarpum* has many chemical constituents, such as chlorogenicacids, stigmasteryl glucoside, glucoalkaloidsolanocarpine, isochlorogenic, carpesterol, methyl ester of 3,4-dihydroxycinnamic acid, neochlorogenic cholesterol, 3,4-dihydroxycinnamicacid(caffeicacid), solanine-S, solasodine, Quercetin 3-O-D-Glucopyranosyl-(1,6)-D-Glucopyranoside, solasonine, Sitosterol-beta-D-Galactoside, solasurine, solamargine, cycloartanol, sitosteryl-glucoside, campesterol, stigmasterol(fruit); sitosterol, flavonal glycoside, apigenin (flower); amino acids and solanocarpine (seeds); esculetin, coumarins, esculin, scopolin and scopoletin (leaves, fruits androots); norcarpesterol, tomatidenolandcarpesterol (plant) [116]. Supplementary materials Figures S3–S5 show their biological mechanism and their MIC 50 values are explained in Table 2. Previous researches have also shown that Kantakari fruit juice shows complete growth inhibition of anti-viral (HIV), anticancer, and anti-mycobacterial activities [117].

## 7. Conclusions

Natural chemicals can be utilized to enhance the efficacy of anti-tuberculosis treatments and perhaps fill in the gaps where regular prescription therapies have lost their effectiveness. Prevention and treatment strategies that combine natural substances may be feasible alternatives for reducing drug resistance. As discussed, natural substances possess a multitude of anti-mycobacterial characteristics and focus on several therapeutic targets. For instance, natural compounds can augment the sensitivity of mycobacterium to antibiotic treatment. Natural items should be researched further for the treatment of active TB. It is worth noting that many of the studies included in this review were carried out using techniques such as molecular assays, mouse models, animal cells, and bacterial culture. Natural products must be authentic, well-formulated, regularly derived from their sources, of excellent quality, and not contaminated with other products. Novel natural chemicals are being researched in the hope that they can be effective in treating tuberculosis infections.

We emphasize identifying plants based on ethnomedical complaints and testing their extracts/phytomolecules against *Mycobacterium paratuberculosis* strains. In conclusion, we attempted to provide a brief idea about the natural compounds which could be suitable for paraphrased research activity against *paratuberculosis*. As a result, we can assume that two plants in particular (*Ocimum sanctum* and *Solanum xanthocarpum*) could achieve a good combinational effect, although any antagonistic effect has yet to be determined. Therefore, targeting these two agents in the future could help shorten the current therapeutic regimens for *paratuberculosis* (paraTB) and aid treatment in other tuberculosis diseases.

## Figures and Tables

**Table 1:** Biological Mechanism between Bioactive constituents with MIC_50_ Values.

S.No.	Bioactive Constituents	MIC_50_ Value	Mechanism	References
1	Eugenol	500 μg/ml	Antifungal	Ahmad et al., 2015 [102]
2	Linalool	0.12%	Antimicrobial	Federman et al., 2016 [103]
3	Ursolic acid	12 μg/mL	Anti-MAP	Navabharat M et al., 2022 [104]
4	beta-caryophyllene	32 μg/mL 1024 μg/ml	Antimicrobial	Santos et al., 2021 [105]
5	Propionic acid	0.25% 0.125%	Antimicrobial Antifungal	Haque et al., 2009 [106]
6	Rosmarinic acid	1.2 mg/mL 0.3 mg/mL 2.5 mg/ml	Antimicrobial Antifungal Antiviral	Abedini et al., 2013 [107]
7	Apigenin	>4 mg/ml	Antimicrobial	Nayaka et al., 2014 [108]
8	Orientin	500 μg/mL 1000 μg/ml	Antimicrobial	Karpiński et al., 2019 [109]
9	Isothymusin	200 μg/mL	Antimicrobial	https://www.chemfaces.com/natural/Isothymusin-CFN97562.html, accessed on 5 July 2022 [110]
10	Vicenin-2	>188 μg/mL	Antimicrobial Antifungal	Mohotti et al., 2020 [111]

**Table 2:** Biological mechanism between Bioactive constituents with MIC 50 Values.

S.No.	Bioactive Constituents	MIC_50_ Value	Mechanism	References
1	Chlorogenic Acid	20 to 80 μg/mL	Antibacterial	Lou et al., 2011 [118]
2.	Stigmasterolglucoside	0.67 mg/ml	Antibacterial	Swain and Padhy et al., 2015 [119]
3.	3,4-dihydroxy cinnamic acid methyl ester	50–200 μg/mL	Antibacterial	Hua Du1 et al., 2009 [120]
4.	Solasodine	60 μg/mL	Anti-MAP	Navabharath M et al., 2022 [104]
5.	Solanine	240 μg/mL 120 μg/mL 90 μg/mL	Antifungal Antiviral Antibacterial	Kumar P et al., 2009 [121]
6.	Cycloartanol	8 µg/mL	Antibacterial	Woldemichael et al., 2004 [122]
7.	Stigmesterol	3.13 μg/mL 6.25 μg/mL	Antibacterial	Mailafiya et al., 2018 [123]
8.	Beta-Sitosterol	6.25 µg/mL 12.5 µg/ml	Antibacterial	NWEZE et al., 2019 [124]
9.	Apigenin	>4 mg/mL	Antibacterial	Nayaka et al., 2014 [108]
10.	Esculestin	192 mg/mL <0.015625 μg/mL	Antibacterial Antifungal	Yang et al., 2016 [125]
11	Esculin	2500 mg/L 625 mg/L	Antibacterial Antifungal	Mokdad-Bzeouich et al., 2014 [126]
12.	Scopoletin	50 μg/mL (without sorbitol) >200 μg/mL (with sorbitol)	Antifungal	Lemos et al., 2020 [127]

## Data Availability

Data are contained within the article.

## Supplementary Materials

The following supporting information can be downloaded at: https://www.mdpi.com/article/10.3390/molecules28083490/s1, Figure S1. Structures and IUPAC names of 1–9 bio molecules of Ocimum sanctum plant. Figure S2. Structures and IUPAC names of 10–12 bio molecules of Ocimum sanctum plant. Figure S3. Structures and IUPAC names of 1–8 bio molecules of Solanum xanthocarpum plant. Figure S4. Structures and IUPAC names of 9–16 bio molecules of Solanum xanthocarpum plant. Figure S5. Structures and IUPAC names of 17–20 bio molecules of Solanum xanthocarpum plant.
