# Supplementary material for: Bioprospective Role of Ocimum sanctum and Solanum xanthocarpum against Emerging Pathogen: Mycobacterium avium Subspecies paratuberculosis: A Review
Source: Molecules. 2023 Apr 15;28(8):3490. doi: 10.3390/molecules28083490 (PMC10145132; doi:10.3390/molecules28083490)
Supplement: Supplementary file 1 [file molecules-28-03490-s001.zip › molecules-2305878-supplementary.pdf]

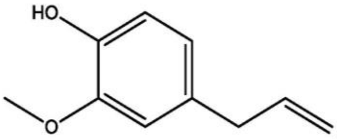

2-methoxy-4-prop-2-enylphenol

1. Eugenol

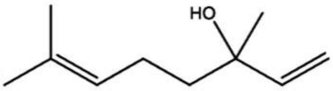

3,7-dimethylocta-1,6-dien-3-ol

2. Linalool

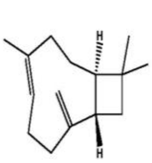

(1R,4E,9S)-4,11,11-trimethyl-8-methylidenebicyclo[7.2.0]undec-4-ene

3. Beta-caryophyllene

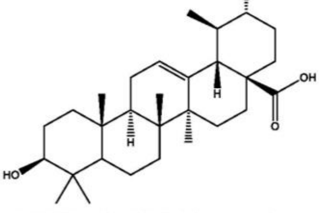

(1*S*,2*R*,4a*S*,6a*S*,6b*R*,10*S*,12a*R*,12b*R*,14b*S*)-10-hydroxy-1,2,6a,6b,9,9,12a-heptamethyl-1,2,3,4,4a,5,6,6a,6b,7,8,8a,9,10,11,12,12a,12b,13,14b-icosahydropicene-4a-carboxylic acid

4. Urosolic acid

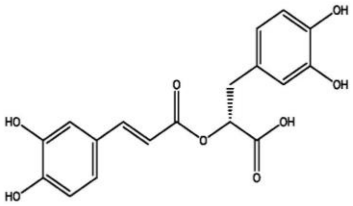

(2R)-3-(3,4-dihydroxyphenyl)-2-[(E)-3-(3,4-dihydroxyphenyl)prop-2-enoyl]oxypropanoic acid

5. Rosmarinic acid

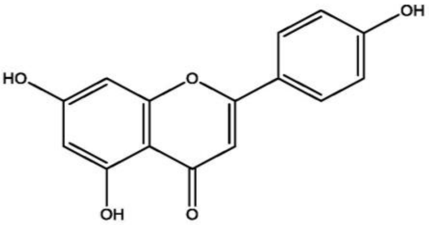

5,7-dihydroxy-2-(4-hydroxyphenyl)chromen-4-one

6. Apigenin

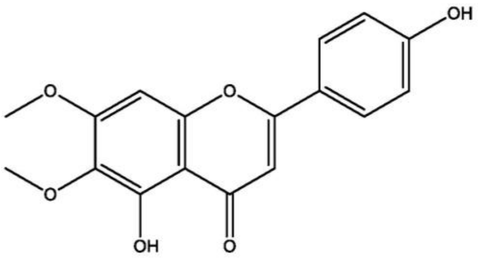

5-hydroxy-2-(4-hydroxyphenyl)-6,7-dimethoxychromen-4-one

7. Cirsimaritin

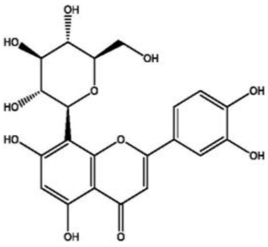

2-(3,4-dihydroxyphenyl)-5,7-dihydroxy-8-[(2S,3R,4R,5S,6R)-3,4,5-trihydroxy-6-(hydroxymethyl)oxan-2-yl]chromen-4-one

8. Orientin

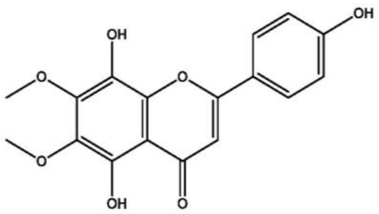

5,8-dihydroxy-2-(4-hydroxyphenyl)-6,7-dimethoxychromen-4-one

9. Isothymusin

Figure S1. Structures and IUPAC names of 1–9 bio molecules of *Ocimum sanctum* plant.

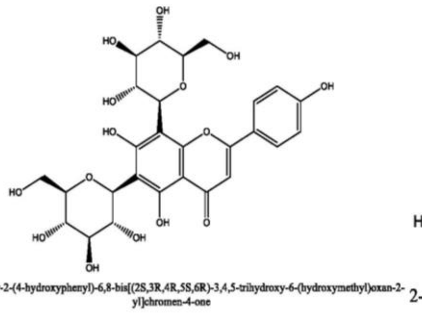

5,7-dihydroxy-2-(4-hydroxyphenyl)-6,8-bis[(2S,3R,4R,5S,6R)-3,4,5-trihydroxy-6-(hydroxymethyl)oxan-2-yl]chromen-4-one

10. Vicenin

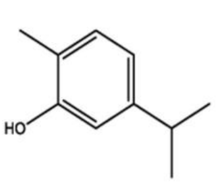

2-methyl-5-propan-2-ylphenol

11. Carvacrol

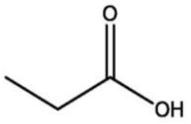

12. Propanoic acid

Figure S2. Structures and IUPAC names of 10–12 bio molecules of *Ocimum sanctum* plant.

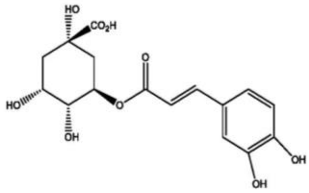

(1*S*,3*R*,4*R*,5*R*)-3-(((*E*)-3-(3,4-dihydroxyphenyl)acryloyl)oxy)-1,4,5-trihydroxycyclohexanecarboxylic acid

1. Chlorogenic acid (CGA)

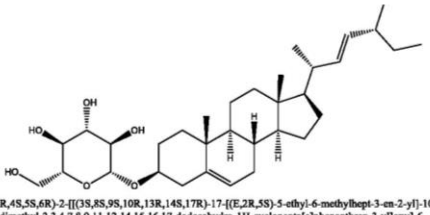

(2R,3R,4S,5S,6R)-2-[[(3S,8S,9S,10R,13R,14S,17R)-17-[(E,2R,5S)-5-ethyl-6-methylhept-3-en-2-yl]-10,13-dimethyl-2,3,4,7,8,9,11,12,14,15,16,17-dodecahydro-1H-cyclopenta[a]phenanthren-3-yl]oxy]-6-(hydroxymethyl)oxane-3,4,5-triol

2. Stigmasterol glucoside

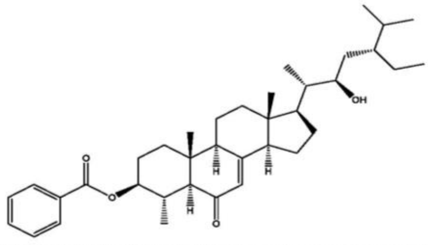

[(3S,4S,5S,9R,10R,13R,14R,17R)-17-[(2S,3R,5R)-5-ethyl-3-hydroxy-6-methylheptan-2-yl]-4,10,13-trimethyl-6-oxo-1,2,3,4,5,9,11,12,14,15,16,17-dodecahydrocyclopenta[a]phenanthren-3-yl] benzoate

3. Carpesterol

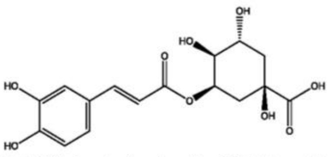

(1R,3R,4S,5R)-3-[(E)-3-(3,4-dihydroxyphenyl)prop-2-enoyl]oxy-1,4,5-trihydroxycyclohexane-1-carboxylic acid

4. Neochlorogenic acid

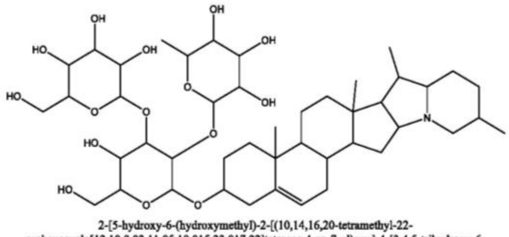

2-[5-hydroxy-6-(hydroxymethyl)-2-[(10,14,16,20-tetramethyl-22-azahexacyclo[12.10.0.02,11.05,10.015,23.017,22]tetracos-4-en-7-yl)oxy]-4-[3,4,5-trihydroxy-6-(hydroxymethyl)oxan-2-yl]oxyoxan-3-yl]oxy-6-methyloxane-3,4,5-triol

5. Solanine

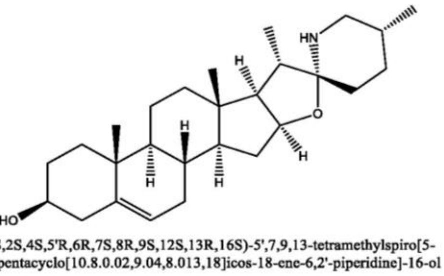

(1S,2S,4S,5'R,6R,7S,8R,9S,12S,13R,16S)-5',7,9,13-tetramethylspiro[5-oxapentacyclo[10.8.0.02,9.04,8.013,18]icos-18-ene-6,2'-piperidine]-16-ol

6. Solasodine

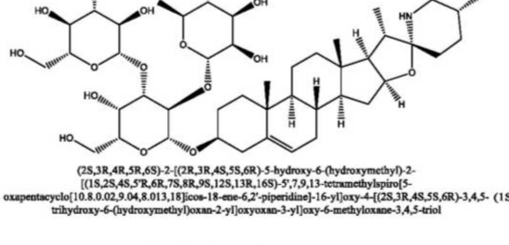

(2S,3R,4R,5R,6S)-2-[(2R,3R,4S,5S,6R)-5-hydroxy-6-(hydroxymethyl)-2-[(1S,2S,4S,5'R,6R,7S,8R,9S,12S,13R,16S)-5',7,9,13-tetramethylspiro[5-oxapentacyclo[10.8.0.02,9.04,8.013,18]icos-18-ene-6,2'-piperidine]-16-yl]oxy-4-[(2S,3R,4S,5S,6R)-3,4,5-trihydroxy-6-(hydroxymethyl)oxan-2-yl]oxyoxan-3-yl]oxy-6-methyloxane-3,4,5-triol

7. Solasonine

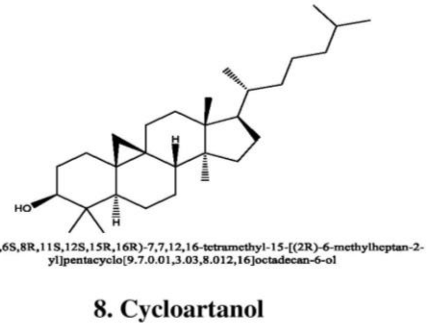

(1S,3R,6S,8R,11S,12S,15R,16R)-7,7,12,16-tetramethyl-15-[(2R)-6-methylheptan-2-yl]pentacyclo[9.7.0.01,3.03,8.012,16]octadecan-6-ol

8. Cycloartanol

Figure S3. Structures and IUPAC names of 1–8 bio molecules of *Solanum xanthocarpum* plant.

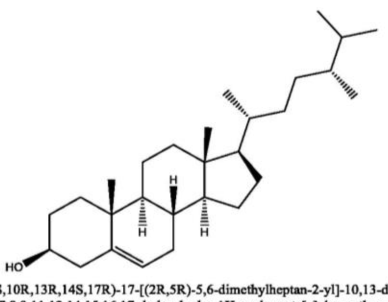

(3S,8S,9S,10R,13R,14S,17R)-17-[(2R,5R)-5,6-dimethylheptan-2-yl]-10,13-dimethyl-2,3,4,7,8,9,11,12,14,15,16,17-dodecahydro-1H-cyclopenta[a]phenanthren-3-ol

9. Campesterol

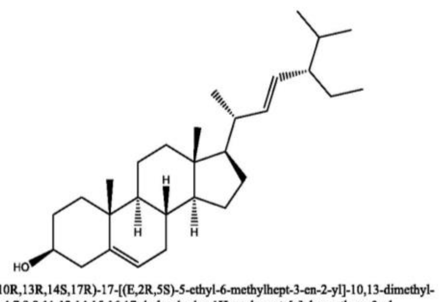

(3S,8S,9S,10R,13R,14S,17R)-17-[(E,2R,5S)-5-ethyl-6-methylhept-3-en-2-yl]-10,13-dimethyl-2,3,4,7,8,9,11,12,14,15,16,17-dodecahydro-1H-cyclopenta[a]phenanthren-3-ol

10. Stigmasterol

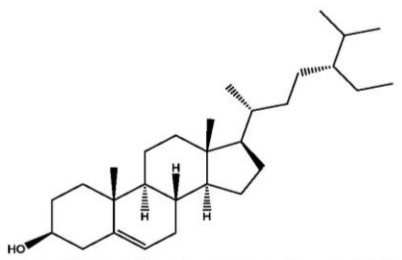

(3S,8S,9S,10R,13R,14S,17R)-17-[(2R,5R)-5-ethyl-6-methylheptan-2-yl]-10,13-dimethyl-2,3,4,7,8,9,11,12,14,15,16,17-dodecahydro-1H-cyclopenta[a]phenanthren-3-ol

11. Beta-sitosterol

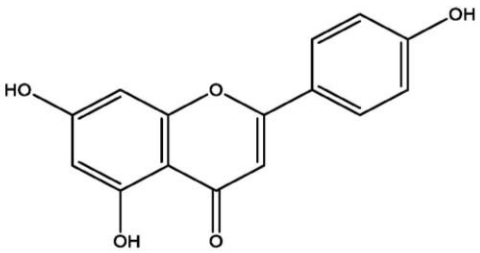

5,7-dihydroxy-2-(4-hydroxyphenyl)chromen-4-one

12. Apigenin

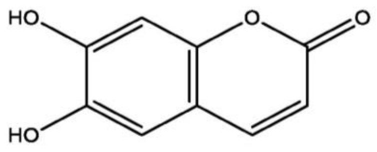

6,7-dihydroxychromen-2-one

13. Esculetin

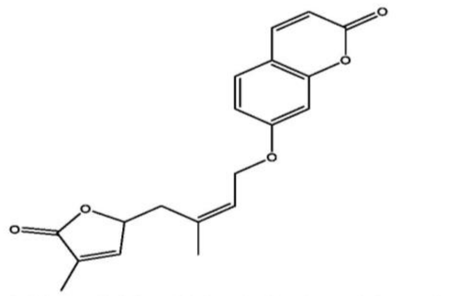

7-[(Z)-3-methyl-4-(4-methyl-5-oxo-2H-furan-2-yl)but-2-enoxy]chromen-2-one

14. Coumarin

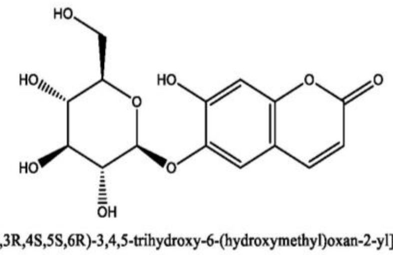

7-hydroxy-6-[(2S,3R,4S,5S,6R)-3,4,5-trihydroxy-6-(hydroxymethyl)oxan-2-yl]oxychromen-2-one

15. Esculin

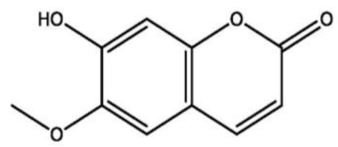

7-hydroxy-6-methoxychromen-2-one

16. Scopoletin

Figure S4. Structures and IUPAC names of 9–16 bio molecules of *Solanum xanthocarpum* plant.

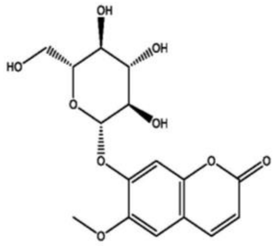

6-methoxy-7-[(2S,3R,4S,5S,6R)-3,4,5-trihydroxy-6-(hydroxymethyl)oxan-2-yl]oxychromen-2-one

17. Scopolin

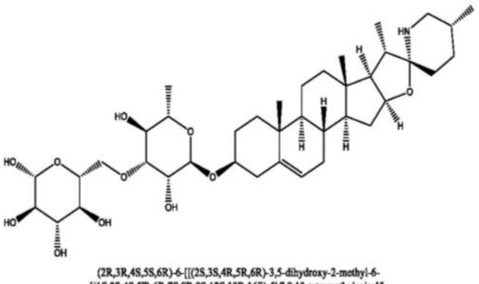

(2R,3R,4S,5S,6R)-6-[[(2S,3S,4R,5R,6R)-3,5-dihydroxy-2-methyl-6-[(1S,2S,4S,5'R,6R,7S,8R,9S,12S,13R,16S)-5',7,9,13-tetramethylspiro[5-oxapentacyclo[10.8.0.02,9.04,8.013,18]icos-18-ene-6,2'-piperidine]-16-yl]oxyoxan-4-yl]oxymethyl]oxane-2,3,4,5-tetrol

18. Solasurine

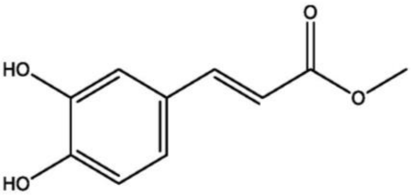

methyl 3-(3,4-dihydroxyphenyl)prop-2-enoate

19. 3,4-dihydroxycinnamic acid methyl ester

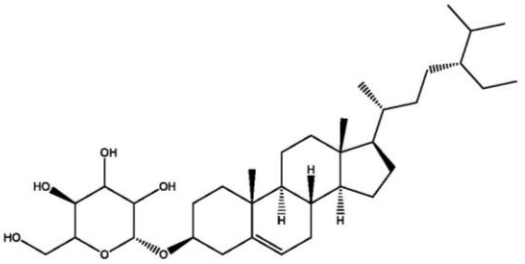

(2R,5S)-2-[[(3S,8S,9S,10R,13R,14S,17R)-17-[(2R,5R)-5-ethyl-6-methylheptan-2-yl]-10,13-dimethyl-2,3,4,7,8,9,11,12,14,15,16,17-dodecahydro-1H-cyclopenta[a]phenanthren-3-yl]oxy]-6-(hydroxymethyl)oxane-3,4,5-triol

20. Sitosteryl-glucoside

Figure S5. Structures and IUPAC names of 17–20 bio molecules of *Solanum xanthocarpum* plant.
